# Circulating Angiogenic Factors and the Risk of Adverse Outcomes among Haitian Women with Preeclampsia

**DOI:** 10.1371/journal.pone.0126815

**Published:** 2015-05-12

**Authors:** Melissa I. March, Carl Geahchan, Julia Wenger, Nandini Raghuraman, Anders Berg, Hamish Haddow, Bri Ann Mckeon, Rulx Narcisse, Jean Louis David, Jennifer Scott, Ravi Thadhani, S. Ananth Karumanchi, Sarosh Rana

**Affiliations:** 1 Department of Obstetrics and Gynecology, Beth Israel Deaconess Medical Center, Boston, MA, United States of America; 2 Division of Maternal Fetal Medicine/Department of Obstetrics and Gynecology, Beth Israel Deaconess Medical Center and Harvard Medical School, Boston, MA, United States of America; 3 Center for Vascular Biology and Research, Beth Israel Deaconess Medical Center Boston, MA, United States of America; 4 Division of Nephrology/Department of Medicine, Massachusetts General Hospital and Harvard Medical School, Boston, MA, United States of America; 5 Department of Pathology, Beth Israel Deaconess Medical Center and Harvard Medical School, Boston, MA, United States of America; 6 Savjani Institute for Health Research, Windham, ME, United States of America; 7 Department of Obstetrics and Gynecology, Hospital Albert Schweitzer, Deshapelles, Haiti; 8 Division of Women’s Health/Department of Medicine, Brigham and Women’s Hospital, Boston, MA, United States of America; 9 Division of Nephrology/Department of Medicine, Beth Israel Deaconess Medical Center, and Harvard Medical School, Boston, MA, United States of America; 10 Howard Hughes Medical Institute, Boston, MA, United States of America; 11 Maternal Fetal Medicine/Department of Obstetrics and Gynecology, University of Chicago, Chicago, IL, United States of America; University of Barcelona, SPAIN

## Abstract

**Objective:**

Angiogenic factors are strongly associated with adverse maternal and fetal outcomes among women with preterm preeclampsia (PE) in developed countries. We evaluated the role of angiogenic factors and their relationship to adverse outcomes among Haitian women with PE.

**Material and Methods:**

We measured plasma antiangiogenic soluble fms-like tyrosine kinase 1 (sFlt1) and proangiogenic placental growth factor (PlGF) levels in women with PE (n=35) compared to controls with no hypertensive disorders (NHD) (n=43) among subjects with singleton pregnancies that delivered at Hospital Albert Schweitzer (HAS) in Haiti. We divided the preeclamptic women into two groups, early onset (≤ 34 weeks) and late onset (>34 weeks) and examined relationships between sFlt1/PlGF ratios on admission and adverse outcomes (abruption, respiratory complications, stroke, renal insufficiency, eclampsia, maternal death, birth weight <2500 grams, or fetal/neonatal death) in women with PE subgroups as compared to NHD groups separated by week of admission. Data are presented as median (25th-75th centile), n (%), and proportions.

**Results:**

Among patients with PE, most (24/35) were admitted at term. Adverse outcome rates in PE were much higher among the early onset group compared to the late onset group (100.0% vs. 54.2%, P=0.007). Plasma angiogenic factors were dramatically altered in both subtypes of PE. Angiogenic factors also correlated with adverse outcomes in both subtypes of PE. The median sFlt1/PlGF ratios for subjects with early onset PE with any adverse outcome vs. NHD <=34 weeks with no adverse outcome were 703.1 (146.6, 1614.9) and 9.6 (3.5, 58.6); P<0.001). Among late onset group the median sFlt1/PlGF ratio for women with any adverse outcome was 130.7 (56.1, 242.6) versus 22.4 (10.2, 58.7; P=0.005) in NHD >34 weeks with no adverse outcome.

**Conclusion:**

PE-related adverse outcomes are common in women in Haiti and are associated with profound angiogenic imbalance regardless of gestational age at presentation.

## Introduction

Preeclampsia (PE) is a common hypertensive complication of pregnancy and is a leading cause of maternal and fetal morbidity and mortality, especially in developing countries [1]. Preeclampsia is often insidious in its onset and can affect many organ systems, including kidney, liver, heart and brain [2]. Thus, if not diagnosed early and treated promptly, serious complications including death can follow. Routine prenatal care including blood pressure (BP) and urine dipstick evaluations as practiced in developed nations has resulted in early detection of PE, often followed by timely delivery and avoiding the most serious outcomes [2]. In fact, in developed nations PE is the leading cause of iatrogenic prematurity, in a sense shifting the burden of disease to the neonate [3]. On the other hand, the depiction of PE and its outcomes differs between developed and developing nations. While in developed nations the majority of PE-related adverse outcomes occur among in the early onset group (gestational ages, GA, <34 weeks) the overall prevalence of early onset preeclampsia is very low. However, in developing nations, likely due to the failure of early detection of the disorder, the majority of patients present at term and with high rates of adverse outcomes [4, 5].

There is considerable evidence that PE phenotypes are related to abnormal circulating angiogenic factors, specifically an elevation of anti-angiogenic factors (Soluble fms like tyrosine kinase- sFlt1) and a decrease in pro-angiogenic factors (Placental growth factor PlGF) [6–12]. In a recent study examining the prediction of PE related adverse outcomes, we have shown that these factors can predict adverse outcomes when measured within 2 weeks of the event; however our data was only significant for GA at presentation of <34 weeks [13]. Others have reported similar findings [10, 14, 15]. This could have two explanations; there could be different subtypes of PE related adverse outcomes—one that occurs preterm and is associated with angiogenic imbalance and another that occurs only at term and is unrelated to angiogenic factor abnormalities [12]. Or it may be that because of nearly universal prenatal care in developed countries and constant surveillance for hypertension, PE gets diagnosed earlier in pregnancy and only patients with mild disease are permitted to progress to term, the natural history of the disease therefore modified by the intervention along the way.

The present study was designed in rural Haiti where data from our recent retrospective study at the same hospital, showed that the prevalence of PE and its related adverse outcomes is high [16]. We measured pre-delivery angiogenic factors and recorded PE related adverse outcomes, hypothesizing that the majority of adverse outcomes related to PE would be associated with angiogenic imbalance, regardless of GA.

## Material and Methods

This study was approved by the Beth Israel Deaconess Medical Center institutional review board (IRB) and by the Hospital Albert Schweitzer board of directors. Because discarded blood samples were used for analysis, informed patient consent was not required for this study. Institutional review board waived the need for written informed consent from the participants for both the use of discarded blood samples and review of medical records.

### Study Design

This study was conducted at Hospital Albert Schweitzer (HAS), located in Deschapelles, Haiti, about 90 miles north of Port-au-Prince, which performs 800–900 deliveries a year. The study was conducted from September 2013 to March 2014 and discarded blood samples collected as part of routine clinical care were used for analysis. For this study, singleton pregnancies with a diagnosis of antepartum PE or antepartum eclampsia were included, if they had both an antepartum blood sample and a recorded GA at admission. Patients with normal BP’s who delivered during the same time period (and had an available blood sample) were included as controls (No Hypertensive Disorder- NHD). All patients undergoing scheduled cesarean delivery and many patients admitted for uncomplicated labor as well as non-hypertensive pregnancy complications (such as preterm labor) generally had a blood sample drawn on admission. Patients with twins, postpartum PE, postpartum eclampsia, gestational hypertension, chronic hypertension and superimposed PE were excluded. Patients were divided into early onset PE (admitted at ≤34 weeks) and late onset PE (>34 weeks). Pre-delivery plasma samples from the same day as delivery were stored in -20°C for 3 months and then shipped to Boston, MA on dry ice for measurement of plasma angiogenic factors. We reviewed medical records to record past medical and gynecological history, social history, prenatal follow up, vital signs, laboratory values, GA, mode of delivery, birth weight, clinical diagnosis and maternal and neonatal complications and entered into a RedCap database [17] for further analysis. Few patients had documented records of prenatal care available for review. The patient data and samples were de-identified prior to analysis.

### Diagnosis of PE

PE was defined as new onset hypertension (HTN, Blood Pressure ≥ 140/90mmHg) and proteinuria (urine dipstick of ≥ 1+) after 20 weeks of gestation. In the absence of proteinuria, PE was also defined by severe HTN (≥160/110) and laboratory abnormalities consistent with hemolysis, low platelets and elevated liver enzymes (HELLP syndrome) or symptoms (i.e. headache, visual changes, right upper quadrant pain). Antepartum eclampsia was diagnosed in patients with antepartum PE who developed seizures. Renal insufficiency was defined as creatinine ≥ 1.1 mg/dL. The clinical diagnoses of PE were confirmed by study staff.

### PE related adverse outcomes

We recorded preeclampsia related adverse maternal and neonatal outcomes that included placental abruption, maternal respiratory complications (such as pulmonary edema, oxygen desaturation and symptoms such as tachypnea), stroke, renal insufficiency, eclampsia, maternal death, birth weight <2500 grams, fetal or neonatal death. All outcomes were ascertained only in presence of HTN (SBP> = 140 or DBP> = 90). For this study, the adverse outcomes were grouped into 4 categories as:

1. Category I: Severe hypertension, defined as BP≥160/110
2. Category II: Abruption, respiratory complications, stroke, renal failure, birth weight <2500 grams
3. Category III: Eclampsia
4. Category IV: Maternal, fetal or neonatal death
5. Category Composite: any of the above adverse outcomes (Category I to IV)

### Measurement of angiogenic factors

Automated assays for sFlt1 and PlGF were performed with commercially available automated assays on Elecsys platform (Roche Diagnostics) as previously described [13, 18]. The CVs for both these assays were <5%. All measurements were done after delivery on all patients and the treating physicians in Haiti were unaware of the test results of sFlt1 and PlGF values. All de-identified samples were thawed once for analysis and the technician doing the analysis was blinded to the patient’s diagnosis.

### Statistical analysis

Characteristics at presentation of normotensive women (NHD) by weeks at admission and women with early onset PE and late onset PE were analyzed using medians (quartile 1, quartile 3) or n (%, percent), as appropriate. Characteristics of each PE group were compared to controls separately using Mann-Whitney U tests or Chi-squared tests. Frequencies of adverse outcomes categories were compared between early onset and late onset PE groups using Chi-squared tests. Due to the heavily right skewed distribution of sFlt1/PlGF ratio, the mean and standard error of natural log transformed (ln) sFlt1/PlGF ratio was used for visualization in figures. Univariate linear regression models were used to compare ln sFlt1/PlGF between diagnosis groups as well as between adverse outcome categories within early and late onset PE women. Finally, Pearson correlation coefficients were used to measure the degree of linear association between ln sFlt1/PlGF ratio, birth weight, highest SBP, highest DBP, and GA at delivery. All P values were 2 sided, and values of P<0.05 were considered statistically significant. All statistical analyses were performed with SAS version 9.4.

## Results

During the study period, we evaluated 43 patients who delivered with NHD and 35 with PE for plasma angiogenic factor abnormalities. The majority of patients with PE were admitted after 34 weeks (N = 24). The patients with early onset PE were older then NHD at ≤34 weeks. These patients also had higher systolic and diastolic BPs (all P<0.05). History of PE was more common in patients with preterm PE compared to NHD ≤34 weeks, although this difference did not reach statistical significance (P = 0.05). Compared to patients with NHD >34 weeks, patients with late onset PE had higher systolic and diastolic BPs (P<0.05). These latter patients had similar GA at admission and delivery as the controls. There were no other differences among the groups in terms of parity, history of PE or history of chronic HTN. Data on prenatal visits were missing in the majority of patients. The clinical characteristics at presentation of patients are shown in Table 1.

**Table 1:** Clinical presentation of patients in Hospital Albert Schweitzer with diagnosis of antepartum preeclampsia and eclampsia and gestational age.

	No HTN Disorder (≤34 weeks)	Early Onset PE (≤34 weeks)	P-value^1^	No HTN Disorder (>34 weeks)	Late Onset PE (>34 weeks)	P-value^2^
N	8	11		35	24	
Age (years)	22 (20, 27)	34 (25, 42)	0.03*	27 (22, 30)	32 (22, 38)	0.09
Parity			0.31			0.14
Nulliparous	4 (50.0)	3 (27.3)		20 (57.1)	9 (37.5)	
Parous	4 (50.0)	8 (72.7)		15 (42.9)	15 (62.5)	
GA on Admission (weeks)	29.8 (26.1, 33.3)	31.0 (28.0, 32.0)	0.97	38.7 (36.2, 40.6)	38.0 (36.0, 39.4)	0.28
GA at Delivery (weeks)	29.2 (24.0, 33.1)	31.2 (28.9, 32.1)	0.49	39.0 (36.5, 40.9)	38.0 (36.1, 39.6)	0.27
Highest Systolic BP (mmHg)	105 (100, 115)	180 (180, 240)	<0.001*	120 (116, 130)	170 (160, 180)	<0.001*
Highest Diastolic BP (mmHg)	60 (60, 75)	120 (100, 120)	<0.001*	70 (60, 80)	100 (100, 120)	<0.001*
Prenatal Care (>1 prior visit)			0.08			0.08
Yes	0 (0.0)	0 (0.0)		8 (22.9)	9 (37.5)	
No	2 (25.0)	0 (0.0)		0 (0.0)	2 (8.3)	
Missing	6 (75.0)	11 (100.0)		27 (77.1)	13 (54.2)	
History of PE or Chronic HTN	0 (0.0)	4 (36.4)	0.05	1 (2.9)	3 (12.5)	0.15
Preterm Delivery <37 Weeks			0.81			0.18
Yes	7 (87.5)	10 (90.9)		9 (25.7)	9 (37.5)	
No	0 (0.0)	0 (0.0)		22 (62.9)	15 (62.5)	
Missing	1 (12.5)	1 (9.1)		4 (11.4)	0 (0.0)	
Birth weight (g)	1100 (830, 1700)	1530 (1300, 1680)	0.24	3118 (2680, 3310)	2980 (2570, 3200)	0.27
sFlt1 (pg/ml)	3391 (2412, 4918)	12895 (8303, 17414)	0.006*	4378 (2618, 5731)	6304 (3127, 10638)	0.03*
PlGF (pg/ml)	324.6 (101.8, 881.4)	18.3 (9.9, 22.5)	0.001*	206.8 (60.9, 395.7)	82.6 (57.1, 215.6)	0.08
sFlt1/PlGF ratio	9.6 (3.5, 58.6)	703.1 (146.6, 1614.9)	<0.001*	22.4 (10.2, 58.7)	77.0 (18.3, 145.1)	0.03*

HTN = hypertension, GA = gestational age, BP = blood pressure

^1^No HTN disorder (≤34 weeks) vs. early onset PE (≤34 weeks)

^2^No HTN disorder (>34 weeks) vs. late onset PE (>34 weeks)

*Significant at P<0.05

**Fig 1:**
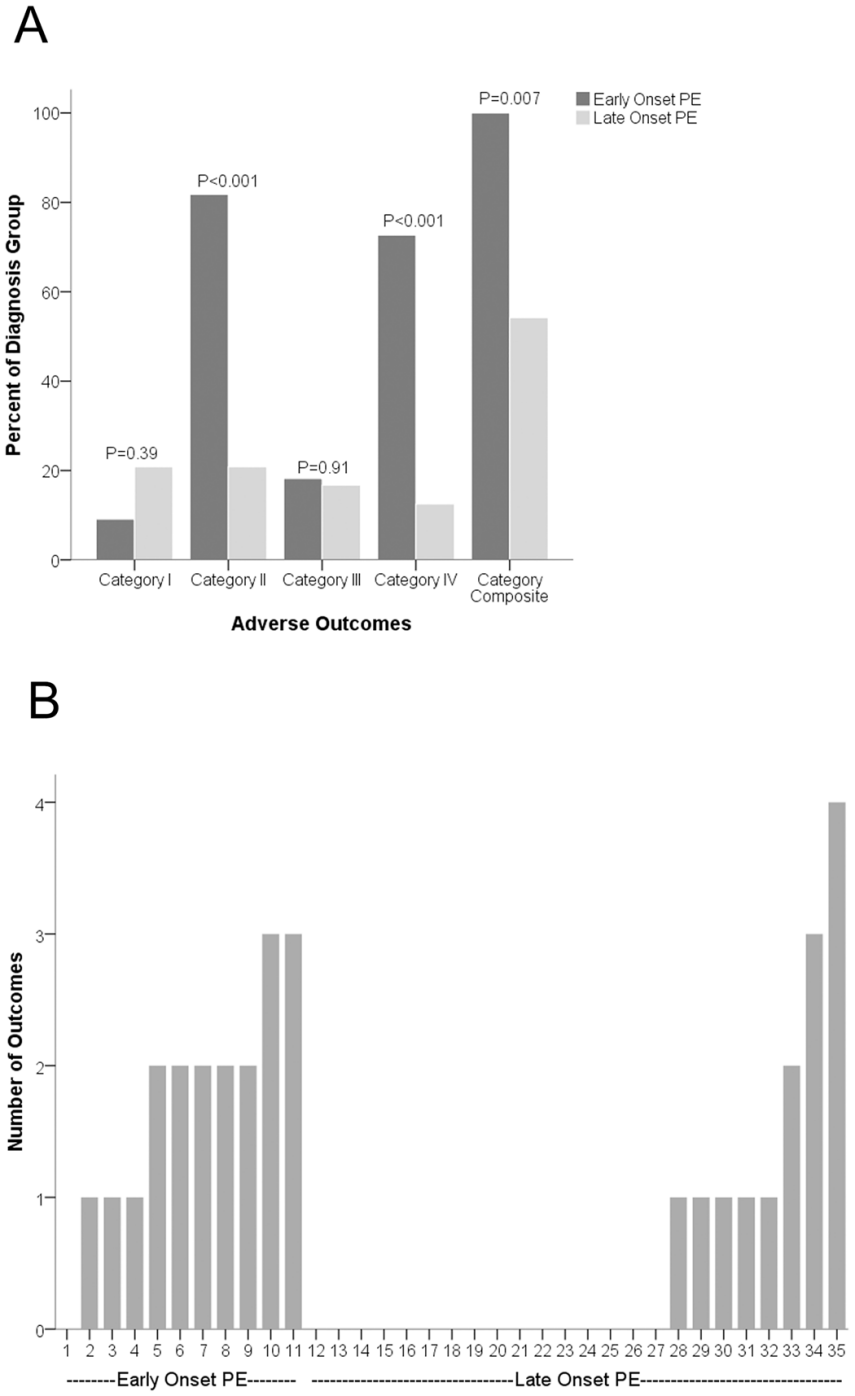
Frequency and number of adverse outcomes among early onset (≤34 weeks) and late onset (>34 weeks) preeclampsia at Hospital Albert Schweitzer in Haiti. (A) Percentage of pregnant patients with early onset and late onset preeclampsia with adverse outcomes (Category I- Severe hypertension (BP ≥160/110), Category II- abruption or respiratory complications or stroke or renal failure or birth weight <2500 grams, Category III- Antepartum Eclampsia, Category IV- Maternal, Fetal and neonatal death, Cat V- any of the above adverse outcomes (Category I to IV)). All outcomes were ascertained only in presence of HTN (SBP> = 140 or DBP> = 90). (B) The x-axis shows the number of adverse outcomes and the y-axis shows each patient among different gestational age groups. Early onset PE = preeclampsia diagnosis at presentation at ≤34 weeks, Late onset PE = preeclampsia diagnosis at presentation at >34 weeks.

Adverse outcomes were noted among patients presenting with both early onset and late onset PE. These outcomes were more common in patients with early onset PE, and in fact were present in 100% of patients with early onset PE. Fig 1A and 1B show the frequency and number of adverse outcomes in patients with PE.

**Fig 2:**
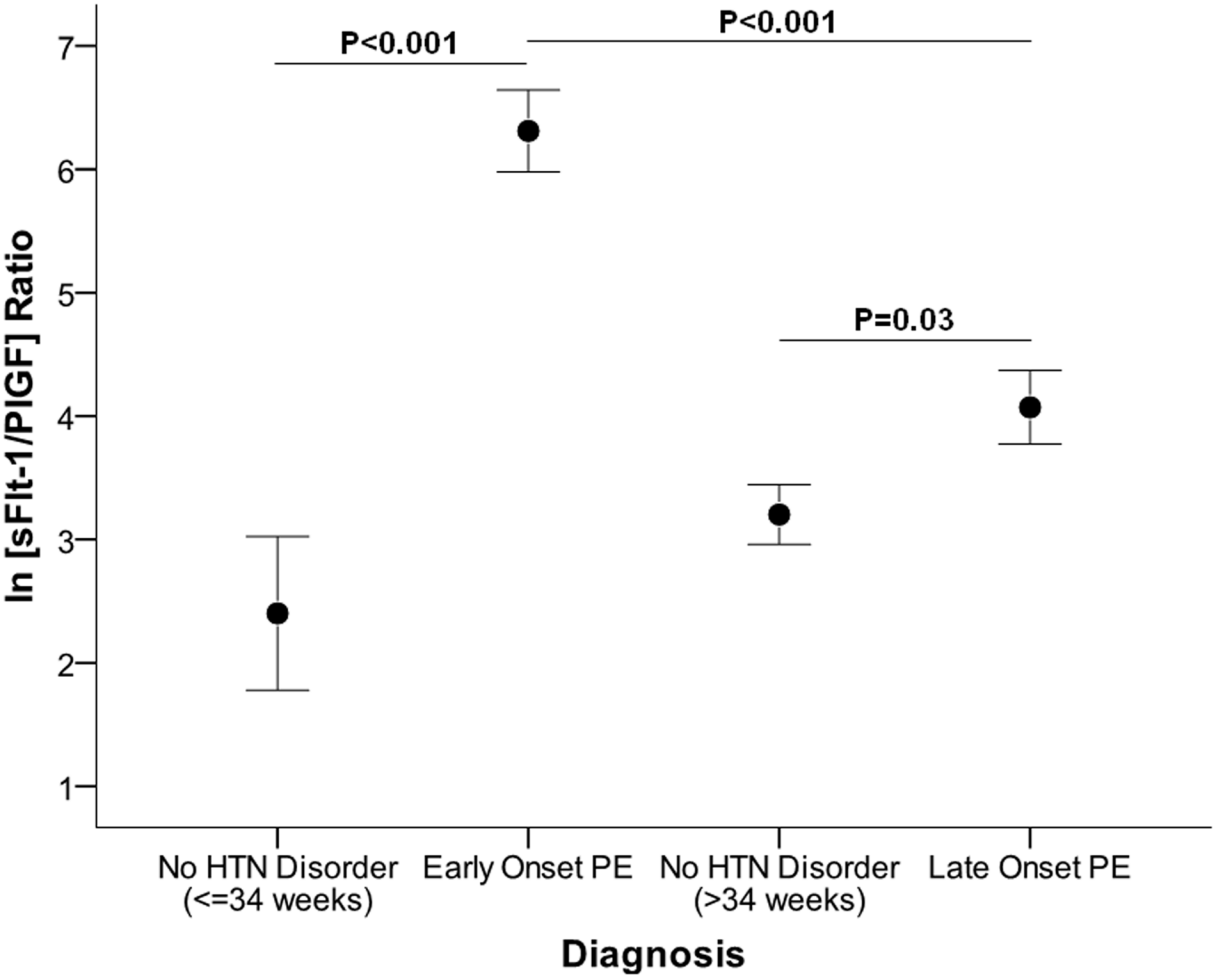
Preeclampsia diagnosis and angiogenic factors at Hospital Albert Schweitzer in Haiti. Ratio of soluble fms-like tyrosine kinase 1 (sFlt1) to placental growth factor (PlGF) at presentation. The distribution of Natural log of sFlt1/PlGF ratio in pregnant women with no hypertensive disorder, early onset preeclampsia (≤34 weeks), and late onset preeclampsia (>34 weeks). Diagnoses were ascertained at the time of presentation. PE = preeclampsia, HTN = hypertension.

Angiogenic factors were altered in patients with the diagnosis of early onset (median sFlt1/PlGF ratio 703.1 (146.6, 1614.9) vs NHD (≤34 weeks) 9.6 (3.5, 58.6), P<0.001) and late onset PE 77.0 (18.3, 145.1) vs. NHD (>34 weeks) 22.4 (10.2, 58.7); P = 0.03). Patients with late onset PE had lower levels of sFlt1/PlGF ratio compared to early onset PE (P<0.001), Table 1 and Fig 2.

**Fig 3:**
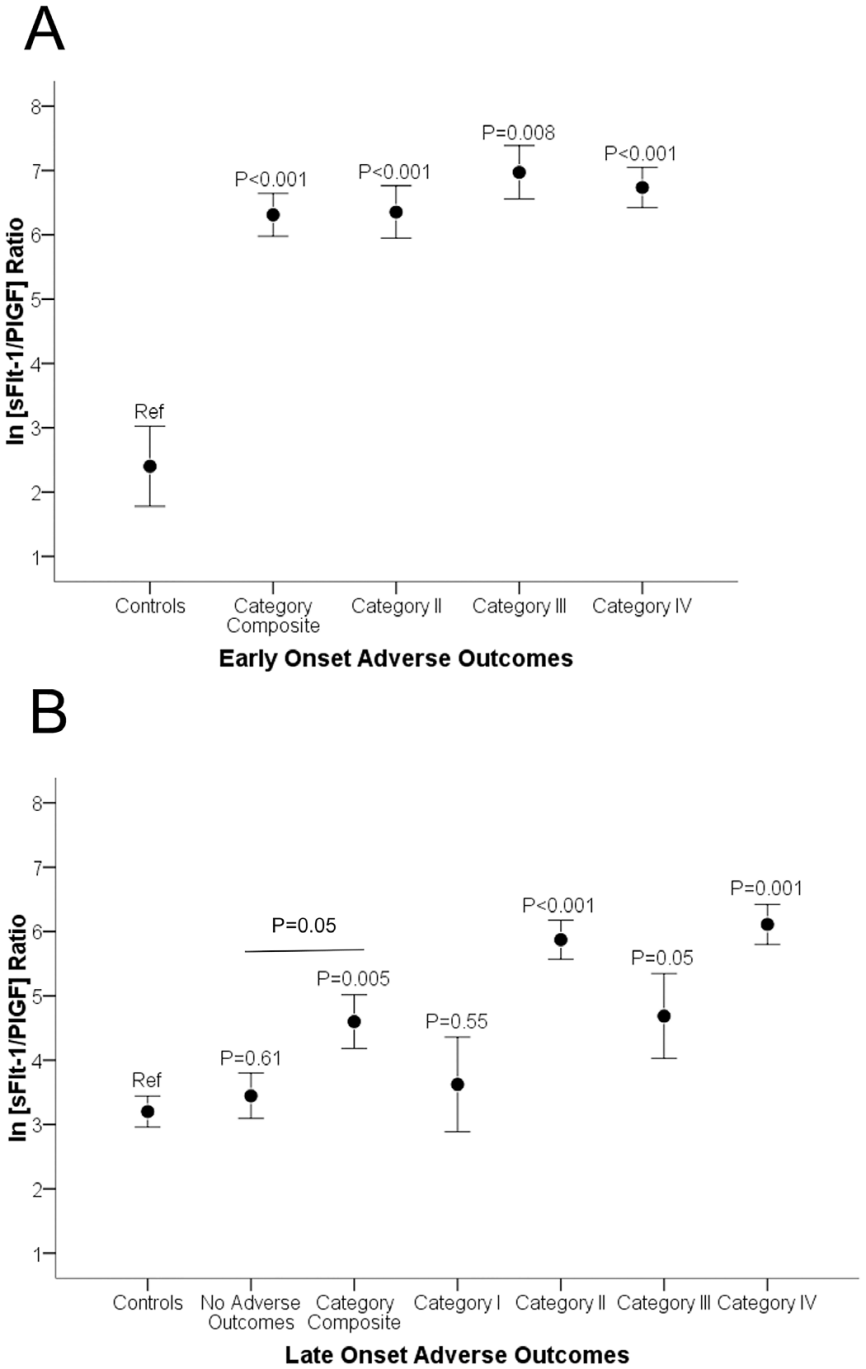
Adverse outcomes and angiogenic factors among patients with preeclampsia at Hospital Albert Schweitzer in Haiti. The distribution of natural log transformed sFlt1/PlGF ratios at initial presentation by adverse outcomes is shown. Category I- Severe hypertension (BP ≥160/110), Category II- abruption or respiratory complications or stroke or renal failure or birth weight <2500 grams, Category III- Antepartum Eclampsia, Category IV- Maternal, Fetal and neonatal death, Cat V- any of the above adverse outcomes (Category I to IV). All outcomes were ascertained only in presence of HTN (SBP> = 140 or DBP> = 90). (A) Distribution among early onset preeclampsia (≤34 weeks). Control = women with no hypertensive disorder with no adverse outcome at ≤34 weeks on admission. (B) Distribution among late onset preeclampsia (>34 weeks). Control = women with no hypertensive disorder with no adverse outcome at >34 weeks on admission. P value compared to controls.

**Fig 4:**
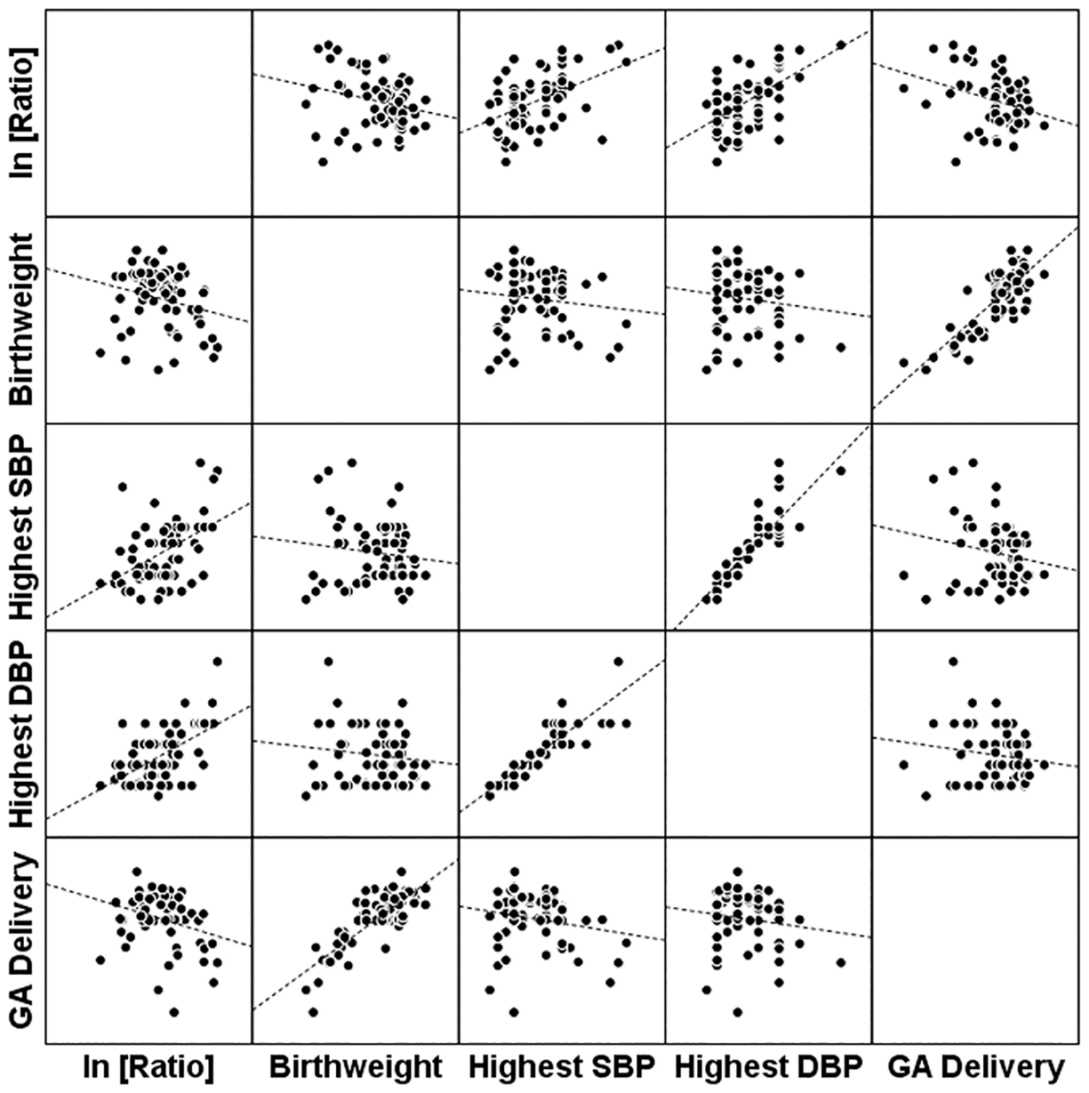
Correlation of angiogenic factor ratio with birth weight, highest systolic blood pressure (SBP) and highest diastolic blood pressure (DBP) and gestational age (GA) at delivery. Pearson correlation with ln (sFlt1/PIGF Ratio) and birth weight (r = -0.25, P = 0.04), highest SBP (r = 0.50, P<0.001), highest DBP (r = 0.57, P<0.001), and GA of delivery (r = -0.30, P = 0.01).

Angiogenic factors also correlated with adverse outcomes. In women < = 34 weeks, the median sFlt1/PlGF values for PE women with any adverse outcome and NHD women with no adverse outcome were 703.1 (146.6, 1614.9) and 9.6 (3.5, 58.6) respectively; P<0.001, (Fig 3A). In women >34 weeks, the median sFlt1/PlGF values for PE women with any adverse outcome and NHD women with no adverse outcome were 130.7 (56.1, 242.6) and 22.4 (10.2, 58.7), respectively; P = 0.005, (Fig 3B). When looking at individual categories, the sFlt1/PlGF ratio was significantly elevated among all categories of adverse outcomes (except severe HTN) among patients with both early onset and late onset PE (Fig 3A and 3B, Table 2). There were no patients without adverse outcomes in early onset PE. Patients with late onset PE with no adverse outcomes had similar levels to patients with no NHD (P = 0.61) and lower levels than patients with any adverse outcome (P = 0.05). The sFlt1/PIGF ratio also correlated with birth weight (r = -0.25, P = 0.04), highest SBP (r = 0.50, P<0.001), highest DBP (r = 0.57, P<0.001), and GA of delivery (r = -0.30, P = 0.01), Fig 4.

**Table 2:** Individual data for angiogenic factors by adverse outcomes of patients with early and late onset preeclampsia.

Diagnosis	Predictors	Values	Controls†	No Adverse Outcomes	Category Composite	Category I	Category II	Category III	Category IV
Early Onset PE	sFlt1 (pg/ml)	N	8	—-	11	—-	9	2	8
		Median (25th-75th centile)	3391 (2412, 4918)	—-	12895* (8303, 17414)	—-	12895* (8303, 17414)	14417* (12895, 15939)	16616* (12272, 17972)
	PlGF (pg/ml)	N	8	—-	11	—-	9	2	8
		Median (25th-75th centile)	324.6 (101.8, 881.4)	—-	18.3* (9.9, 22.5)	—-	13.1* (9.9, 20.2)	14.1* (9.9, 18.3)	15.7* (9.9, 20.8)
	sFlt1/PlGF Ratio	N	8	—-	11	—-	9	2	8
		Median (25th-75th centile)	9.6 (3.5, 58.6)	—-	703.1* (146.6, 1614.9)	—-	891.2* (146.6, 1614.9)	1159.0* (703.1, 1614.9)	911.8* (578.4, 1686.9)
Late Onset PE	sFlt1 (pg/ml)	N	35	11	13	5	5	4	3
		Median (25th-75th centile)	4378 (2618, 5731)	4954 (2606, 7536)	8579* (3877, 14393)	3877 (3549, 5286)	14858* (14393, 15091)	9418* (5520, 12695)	14858* (14393, 15091)
	PlGF (pg/ml)	N	34	11	13	5	5	4	3
		Median (25th-75th centile)	206.8 (60.9, 395.7)	106.9 (78.8, 317.4)	62.2* (35.3, 110.3)	110.3 (46.2, 227.8)	35.3* (24.5, 62.2)	69.5 (40.1, 173.7)	24.5* (23.2, 62.2)
	sFlt1/PlGF Ratio	N	34	11	13	5	5	4	3
		Median (25th-75th centile)	22.4 (10.2, 58.7)	27.4 (8.2, 101.6)	130.7* (56.1, 242.6)	84.0 (15.6, 93.2)	473.1* (242.6, 606.7)	95.2 (42.8, 377.6)	606.7* (242.6, 620.9)

^†^Women with no hypertensive disorder with no adverse outcome

*Significant at P<0.05 compared to NHD (controls)

## Discussion

While PE may have many heterogeneous etiologies, we have hypothesized that adverse maternal and fetal outcomes related to PE are driven by angiogenic imbalance [19]. However while data supporting this hypothesis exists in early onset PE, limited data exists for late onset PE. Because, late onset PE has only modest changes in angiogenic factors, it has been argued that this subtype of PE may be related to a non-angiogenic pathway. In developed countries, PE is modified by constant surveillance and termination of a premature pregnancy by delivery at the earliest sign of an imminent adverse outcome. At term, pregnancy is usually terminated when any sign of possible preeclampsia is noted, whether or not signs of imminent adverse outcomes exist. Therefore there is limited data for women diagnosed at term where relationships between angiogenic factor levels and major adverse outcomes were also assessed [20, 21]. The fact that many preeclamptics in Haiti do not present until later stages of their disease permitted us to make such an assessment.

In this study, performed in a developing nation, we confirmed the angiogenic imbalance in women with a diagnosis of PE similar to previously published data [7, 12]. We also confirmed, as previously reported, that the degree of angiogenic imbalance is less profound at term compared to preterm [12, 22], consistent with the hypothesis that patients with preterm PE have much more severe clinical disease, hence presenting earlier in pregnancy. This is also likely due to the fact that as term approaches, BP rises in all pregnant women and using a cut off for BP for diagnosis of PE may be erroneous. To support the notion that PE is driven by high levels of sFlt1, we found profound angiogenic factor imbalance (high levels of sFlt1 and low levels of PlGF) among all women with PE associated adverse outcomes regardless of GA.

In a previous review [19] we suggested that phenotypic preeclampsia, especially with serious complications, may be contributed by angiogenic factor imbalance, citing evidence such as the use of sFlt1 to create pathological lesions in experimental animals that resembled those in biopsies and autopsies in human preeclampsia [6]. We have also argued that views suggesting other non-angiogenic forms of preeclampsia might also relate to a frequently incorrect diagnosis, as hypertension and proteinuria are non-specific symptoms seen in other disorders, or the fact there is a physiological BP rise towards the end of pregnancy and not all women with hypertension are at risk for adverse outcomes. Our own data show that whichever view is correct, the association of adverse outcomes with angiogenic dysfunction was the form to be identified both for management and appropriate descriptions of the disease.

Our study has several implications. Firstly, it supports our view that most, if not all, PE related adverse outcomes are related to angiogenic imbalance, though further larger studies should be designed in developing countries to validate our findings. If found to be true, this data may be extrapolated to studies done in developed nations. This may indicate that patients with a diagnosis of PE who have a normal angiogenic profile may not need to be delivered preterm in an attempt to prevent adverse outcomes. This is especially true in developing countries where specialized care for premature infants may be hard to find. This will also hold true at term, where currently all women with hypertension (either gestational and PE) are recommended to be delivered at 37 weeks without any assessment of risk of adverse outcomes [23]. If future studies show similar results, it may be that patients with a diagnosis of gestational hypertension or preeclampsia without angiogenic imbalance may be re-classified or managed differently given the low risk of adverse outcomes. Angiogenic factor assessment will also be useful among patients in whom a clear-cut diagnosis of PE with severe features is difficult, such as among patients with lupus nephritis or chronic hypertension. A stratification and management strategy based on angiogenic factors based on recently published cutoffs [24] may help reduce the rate of preterm birth in developed countries by decreasing the number of iatrogenic preterm births indicated due to PE. At the same time, studies need to be done to determine if angiogenic factor measurement is possible in real time and may be helpful in identifying patients who are at high risk of adverse outcomes before the onset of severe morbidity. This will be especially useful in resource limited countries where routine use of prenatal care and identification of asymptomatic disease is limited. These patients can then be transferred to a local hospital for safe delivery, anti-hypertensive treatment and magnesium sulfate for seizure prophylaxis. Data from such settings have shown a reduction of maternal and fetal mortality by timely delivery [25]. This will help reduce the heavy burden of maternal, fetal and neonatal death in these countries.

Our study has several limitations. The data were collected from charts with no effort made to validate clinical or laboratory parameters at HAS. However this was both a pilot and pragmatic study and designed to test the association of the sFlt1/PlGF ratio in a real clinical setting. The availability of both cases and controls was limited by the availability of a discarded blood sample. This likely represents some selection bias, however we believe that most patients with preeclampsia, pregnancy complications and healthy patients had at least one blood sample drawn. We did not record the characteristics of the patients who did not have a blood sample available. Of further note, the blood samples collected were discarded samples and these were processed and stored at -20°C for a period of about 3 months. However angiogenic factors are shown to be stable in plasma or serum when stored in freezer over a prolonged period of time (>10 years) [7]. The study is also limited by the small sample size and a single center experience.

In conclusion, this study adds evidence that PE-related adverse outcomes are largely mediated by angiogenic imbalance, even in term gestations. Further studies need to be done to validate these findings in other rural settings where rates of adverse outcomes are high. More importantly, studies need to be done to evaluate the use of angiogenic factors in risk assessment of women with hypertension in pregnancy for early identification of women at risk for serious morbidity and at the same time of rule out patients not at risk for these outcomes.

## References

[1] Organization WH. World Health Report Make every mother and child count. Geneva 2005

[2] ACOG practice bulletin. Diagnosis and management of preeclampsia and eclampsia. Number 33, January 2002. Obstet Gynecol. 2002;99(1):159–67. 16175681 10.1016/s0029-7844(01)01747-1

[3] Morisaki N, Togoobaatar G, Vogel JP, Souza JP, Rowland Hogue CJ, Jayaratne K, et al Risk factors for spontaneous and provider-initiated preterm delivery in high and low Human Development Index countries: a secondary analysis of the World Health Organization Multicountry Survey on Maternal and Newborn Health. BJOG. 2014;121 Suppl 1:101–9. 10.1111/1471-0528.12631 24641540

[4] Firoz T, Sanghvi H, Merialdi M, von Dadelszen P. Pre-eclampsia in low and middle income countries. Best practice & research Clinical obstetrics & gynaecology. 2011;25(4):537–48. 21592865 10.1016/j.bpobgyn.2011.04.002

[5] Bilano VL, Ota E, Ganchimeg T, Mori R, Souza JP. Risk factors of pre-eclampsia/eclampsia and its adverse outcomes in low- and middle-income countries: a WHO secondary analysis. PloS one. 2014;9(3):e91198 10.1371/journal.pone.0091198 24657964 PMC3962376

[6] Maynard SE, Min JY, Merchan J, Lim KH, Li J, Mondal S, et al Excess placental soluble fms-like tyrosine kinase 1 (sFlt1) may contribute to endothelial dysfunction, hypertension, and proteinuria in preeclampsia. J Clin Invest. 2003;111(5):649–58. 12618519 10.1172/JCI17189 PMC151901

[7] Levine RJ, Maynard SE, Qian C, Lim KH, England LJ, Yu KF, et al Circulating angiogenic factors and the risk of preeclampsia. The New England journal of medicine. 2004;350(7):672–83. 14764923 10.1056/NEJMoa031884

[8] Romero R, Nien JK, Espinoza J, Todem D, Fu W, Chung H, et al A longitudinal study of angiogenic (placental growth factor) and anti-angiogenic (soluble endoglin and soluble vascular endothelial growth factor receptor-1) factors in normal pregnancy and patients destined to develop preeclampsia and deliver a small for gestational age neonate. J Matern Fetal Neonatal Med. 2008;21(1):9–23. 10.1080/14767050701830480 18175241 PMC2587364

[9] Kusanovic JP, Romero R, Chaiworapongsa T, Erez O, Mittal P, Vaisbuch E, et al A prospective cohort study of the value of maternal plasma concentrations of angiogenic and anti-angiogenic factors in early pregnancy and midtrimester in the identification of patients destined to develop preeclampsia. J Matern Fetal Neonatal Med. 2009;22(11):1021–38. 10.3109/14767050902994754 19900040 PMC3427777

[10] Chaiworapongsa T, Romero R, Savasan ZA, Kusanovic JP, Ogge G, Soto E, et al Maternal plasma concentrations of angiogenic/anti-angiogenic factors are of prognostic value in patients presenting to the obstetrical triage area with the suspicion of preeclampsia. J Matern Fetal Neonatal Med. 2011;24(10):1187–207. 10.3109/14767058.2011.589932 21827221 PMC3384532

[11] Romero R, Chaiworapongsa T. Preeclampsia: a link between trophoblast dysregulation and an antiangiogenic state. The Journal of clinical investigation. 2013;123(7):2775–7. 10.1172/JCI70431 23934119 PMC3999621

[12] Noori M, Donald AE, Angelakopoulou A, Hingorani AD, Williams DJ. Prospective study of placental angiogenic factors and maternal vascular function before and after preeclampsia and gestational hypertension. Circulation. 2010;122(5):478–87. 10.1161/CIRCULATIONAHA.109.895458 20644016

[13] Rana S, Powe CE, Salahuddin S, Verlohren S, Perschel FH, Levine RJ, et al Angiogenic factors and the risk of adverse outcomes in women with suspected preeclampsia. Circulation. 2012;125(7):911–9. 10.1161/CIRCULATIONAHA.111.054361 22261192 PMC3319742

[14] Moore AG, Young H, Keller JM, Ojo LR, Yan J, Simas TA, et al Angiogenic biomarkers for prediction of maternal and neonatal complications in suspected preeclampsia. J Matern Fetal Neonatal Med. 2012;25(12):2651–7. 10.3109/14767058.2012.713055 22861812

[15] Chappell LC, Duckworth S, Seed PT, Griffin M, Myers J, Mackillop L, et al Diagnostic accuracy of placental growth factor in women with suspected preeclampsia: a prospective multicenter study. Circulation. 2013;128(19):2121–31. 10.1161/CIRCULATIONAHA.113.003215 24190934

[16] Raghuramana N, March MI, Hacker MR, Modesta MA, Wenger J, Narcissee R, David JL, Scott J, Rana S. Adverse maternal and fetal outcomes and deaths related to preeclampsia and eclampsia in Haiti. Pregnancy Hypertension: An International Journal of Women's Cardiovascular Health. 2014; 4:279–286. 10.1016/j.preghy.2014.09.002 26104817

[17] Harris PA, Taylor R, Thielke R, Payne J, Gonzalez N, Conde JG. Research electronic data capture (REDCap)—a metadata-driven methodology and workflow process for providing translational research informatics support. Journal of biomedical informatics. 2009;42(2):377–81. 10.1016/j.jbi.2008.08.010 18929686 PMC2700030

[18] Verlohren S, Galindo A, Schlembach D, Zeisler H, Herraiz I, Moertl MG, et al An automated method for the determination of the sFlt-1/PIGF ratio in the assessment of preeclampsia. American journal of obstetrics and gynecology. 2010;202(2):161 e1–e11. 10.1016/j.ajog.2009.09.016 19850276

[19] Rana S, Karumanchi SA, Lindheimer MD. Angiogenic factors in diagnosis, management, and research in preeclampsia. Hypertension. 2014;63(2):198–202. 10.1161/HYPERTENSIONAHA.113.02293 24166749 PMC3947285

[20] Chaiworapongsa T, Romero R, Korzeniewski SJ, Kusanovic JP, Soto E, Lam J, et al Maternal plasma concentrations of angiogenic/antiangiogenic factors in the third trimester of pregnancy to identify the patient at risk for stillbirth at or near term and severe late preeclampsia. Am J Obstet Gynecol. 2013;208(4):287 e1–e15. 10.1016/j.ajog.2013.01.016 23333542 PMC4086897

[21] Garcia-Tizon Larroca S, Tayyar A, Poon LC, Wright D, Nicolaides KH. Competing risks model in screening for preeclampsia by biophysical and biochemical markers at 30–33 weeks' gestation. Fetal diagnosis and therapy. 2014;36(1):9–17. 10.1159/000362518 24902880

[22] Levine RJ, Lam C, Qian C, Yu KF, Maynard SE, Sachs BP, et al Soluble endoglin and other circulating antiangiogenic factors in preeclampsia. The New England journal of medicine. 2006;355(10):992–1005. 16957146 10.1056/NEJMoa055352

[23] American College of O, Gynecologists, Task Force on Hypertension in P. Hypertension in pregnancy. Report of the American College of Obstetricians and Gynecologists' Task Force on Hypertension in Pregnancy. Obstetrics and gynecology. 2013;122(5):1122–31. 10.1097/01.AOG.0000437382.03963.88 24150027

[24] Verlohren S, Herraiz I, Lapaire O, Schlembach D, Zeisler H, Calda P, et al New gestational phase-specific cutoff values for the use of the soluble fms-like tyrosine kinase-1/placental growth factor ratio as a diagnostic test for preeclampsia. Hypertension. 2014;63(2):346–52. 10.1161/HYPERTENSIONAHA.113.01787 24166751

[25] Goldenberg RL, Jones B, Griffin JB, Rouse DJ, Kamath-Rayne BD, Trivedi N, et al Reducing maternal mortality from preeclampsia and eclampsia in low-resource countries—what should work? Acta obstetricia et gynecologica Scandinavica. 2014; 94(2):148–55. 10.1111/aogs.12533 25353716

